# Status of Epstein-Barr Virus Coinfection with* Helicobacter pylori* in Gastric Cancer

**DOI:** 10.1155/2017/3456264

**Published:** 2017-03-21

**Authors:** Shyam Singh, Hem Chandra Jha

**Affiliations:** Centre for Biosciences and Biomedical Engineering, Indian Institute of Technology, Indore, India

## Abstract

Epstein-Barr virus is a ubiquitous human herpesvirus whose primary infection causes mononucleosis, Burkett's lymphoma, nasopharyngeal carcinoma, autoimmune diseases, and gastric cancer (GC). The persistent infection causes malignancies in lymph and epithelial cells.* Helicobacter pylori* causes gastritis in human with chronic inflammation. This chronic inflammation is thought to be the cause of genomic instability. About 45%-word population have a probability of having both pathogens, namely,* H. pylori* and EBV. Approximately 180 per hundred thousand population is developing GC along with many gastric abnormalities. This makes GC the third leading cause of cancer-related death worldwide. Although lots of research are carried out individually for EBV and* H. pylori*, still there are very few reports available on coinfection of both pathogens. Recent studies suggested that EBV and* H. pylori* coinfection increases the occurrence of GC as well as the early age of GC detection comparing to individual infection. The aim of this review is to present status on coinfection of both pathogens and their association with GC.

## 1. Introduction

Gastric cancer (GC) or stomach cancer is the fifth most common cancer incident and the third leading cause of cancer-associated mortality, contributing 6.8% of total cancer cases and 8.8% of total cancer-associated death worldwide [1]. An estimated 984,000 new cases (ratio 2 : 1, male versus female) and 841,000 GC-related deaths were accounted in 2013 [2]. Approximately, 77% of GC-related cases and the death occur in developing countries, particularly in Eastern Asia, while 23% occur in developed nations [2]. GC can be divided into 4 types on the basis of appearance in different cell types: (1) adenocarcinoma: within the cells of the innermost lining of the stomach (mucous surface); (2) lymphoma: cancer of the immune system in lymph stomach tissues, very rare; (3) gastrointestinal stromal tumors: stomach epithelial lining tumors in interstitial cells of Cajal, very rare; (4) carcinoid tumors: typically arising in the hormone-producing cells of the stomach. The common histopathological features of gastric malignancies are adenocarcinoma. It accounts for nearly 90% of GC [3]. Adenocarcinomas are further divided into two parts: (1) cardia, the top part of the stomach; (2) noncardia cancers, depending on location in the stomach where they first appear.* H. pylori* is now a well-known and primary cause of GC [4–10], specifically noncardia cancer [11, 12], and is declared as carcinogen I [13] to humans.* H. pylori* is now well known to be linked to stomach cancer in many studies along with EBV [14–18]. Other risk factors for GC include chronic gastritis [9], older age [19], male sex [20, 21], a diet high in salt [22–24], smoking [25, 26], alcohol consumption [27], poorly preserved foods [28], diet low in fruits and vegetables [29], tobacco product [30], pernicious anaemia [31–33], a history of stomach surgery for benign conditions [34], and a family history of stomach cancer [34, 35].

## 2. Clinical Association

GC arises mostly in mucosa, the innermost layer in the stomach, and slowly grows out into the other outer layers [58]. GC grows slowly over many years and rarely shows symptoms and is often unnoticed [59–61].* H. pylori* is a spiral-shaped bacterium that grows in the mucus layer which coats the inside of the human stomach, ultimately causing inflammation in the stomach called gastritis [62]. Further, it turns to ulcers [63, 64], long-lasting anaemia [65–67], and growths in the stomach [68, 69], which are more likely to get cancer.* H. pylori* is mainly spread through contaminated water, food, saliva, or mouth to mouth contacts and possibly transmitted sexually via oral-genital contact [70, 71]. Nearly, 50% of the global population is estimated to be infected by* H. pylori* [70, 72], in which less than 2% develop GC [73]. The bacterium is thought to be first acquired during childhood in all nations [74, 75] and mostly in developing countries. Moreover, the infection rate of children in developing countries is higher than that in the advanced countries, 80% compared to 10%, at the age of 20 years [76], while senior citizens in both types of countries have around 50% of infection at 60 yeasr of age.

**Figure 1:**
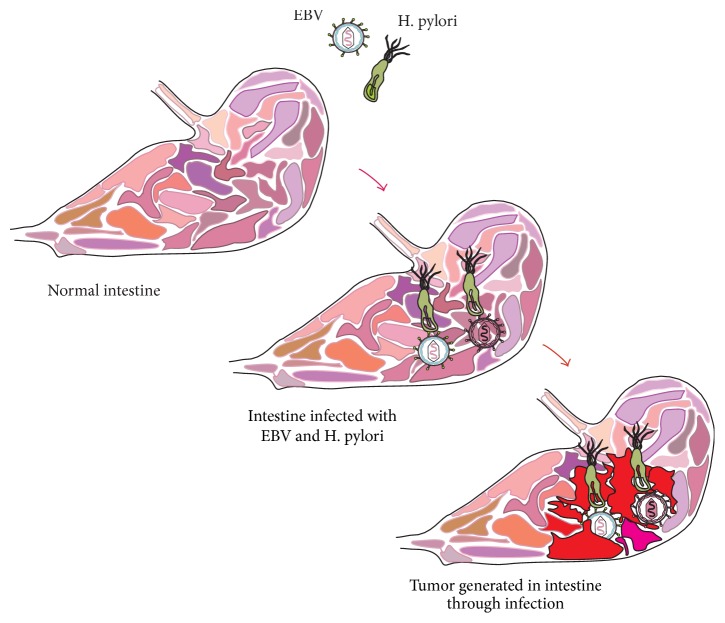
EBV and* H. pylori* coinfection in stomach. Stomach infected with EBV and* H. pylori*. Some of gastric epithelial cells coinfected with EBV and* H. pylori*. Further this coinfection turns into aggressive development of carcinoma.

Moreover, 95% of population have Epstein-Barr virus (EBV) in latent stage [77] and the majority of GC risk increases with* H. pylori* and EBV coinfection [78, 79]. EBV is a *γ*-herpes-virus with genome size of 184 kbp [80]. EBV may initiate mononucleosis in human during the primary infection [77, 81]. EBV spreads mainly by the oral route through contact with saliva [82, 83]; after infection, EBV establishes latent infection that is a virus carrier state, which is of three types (latency I, latency II, and latency III) [84, 85]. During latency, a limited number of viral genes are expressed which maintain the viral episome [86, 87]. EBV infection rates in adult and children vary among nations similar to* H. pylori*. People in underdeveloped countries have much higher infection rates than the developed countries and infections are usually acquired in early childhood [88, 89]. EBV is associated with GC worldwide (11% male, 6% female) and widespread human tumors [15, 90–92]. Some of these tumors are associated with the virus lifestyle and behaviour in the B lymphoid system which is a natural niche of EBV, including B-lymphoproliferative disease [93, 94] in the immunocompromised individual [95], Hodgkin lymphoma [96], Burkitt's lymphoma [97, 98], and a subset of diffuse large B-cell lymphomas [99, 100]. Other tumors occur through viral entry into host's different organ tissues or system. These include nasal T/NK cell lymphoma [101, 102], a group of undifferentiated nasopharyngeal carcinomas (NPC) [103, 104], and gastric carcinomas [91, 92], a tumor type which is linked with chronic* H. pylori* infection through many years.* H. pylori* and EBV account for roughly 80% and 10%, respectively, of GC worldwide. EBV-associated GC is located in the cardia (58%), noncardia (42%) [105], while GC associated with only* H. pylori* is mostly noncardia type of adenocarcinoma [11, 106] (Figure 1).

## 3. EBV Detection Methods

### 3.1. Serological Test

Serological tests for EBV are antibodies specific test with EBV antigens and used to define infection status. Three specific antibodies tests are as follows: (1) Anti-Viral Capsid Antigen (VCA) antibodies IgM and IgG: IgM can be detected in early stage of EBV infection and within 4 to 6 weeks disappears [107–109], while for IgG peaks can be detected within 2 to 4 weeks which decline slightly and remain detectable throughout life [109]. (2) Anti-Early Antigen (EA) antibody IgG: IgG can be detected in the acute stage of infection such as mononucleosis or NPC and it disappears after 3 to 6 months [110]. Detection of Anti-EA IgG represents an active or reactivated EBV infection [111]. Nearly in 20% of people, Anti-EA IgG may be detected for years after resolution of active EBV infection [111, 112]. (3) Anti-EBV Nuclear Antigen (EBNA) 1 antibody, IgG: IgG can be detected after 2 to 4 months of primary EBV infection and remain detectable throughout life [112]. These antibodies tests are helpful to distinguish from acute to a past EBV infection [112, 113]. For example, detection of Anti-VCA IgG and IgM indicates active acute infection if Anti-EBNA 1 is not detected [112], while the detection of Anti-VCA IgG and Anti-EBNA IgG without presence of Anti-VCA IgM represents a past infection [112]. However, sometimes it becomes difficult to conclude when Anti-VCA IgG is detected while Anti-VCA IgM and Anti-EBNA are not. This may be a case of acute, past, or a recent infection [112]. Testing of one more parameter can be included to interpret result correctly, that is, detection of Anti-EA (D) IgG antibodies [114, 115]. During EBV reactivation, Anti-VCA IgG, Anti-EBNA 1, and Anti-EA (D) IgG may be detected simultaneously [112]. The serological results and interpretation are listed in Table 1.

### 3.2. PCR/Real-Time PCR Based Detection

EBV DNA and viral load can be detected by PCR/real-time PCR methods [116]. They are more sensitive and specific than serological methods as EBV immunologic response appears after several days of infections [117–119]. After 15-day onset of onset of EBV infection, 100% of EBV DNA is detectable in plasma [118]. Several reports suggested that EBV DNA is present in almost all carcinoma cells in EBV-positive cases [120]. After primary EBV infection due to immune response, EBV DNA declines slowly in PBMCs, rapidly in plasma or serum, and further 3 to 4 weeks, it becomes undetectable [118, 121]. Interestingly, EBV may remain latent in memory cells for an extended period in blood or take a longer time before it reaches a small, stable stage. Copy number range of 1 to 50 of EBV DNA may be detected in a healthy person infected with EBV in white blood cells (WBC) [122]. PCR and real-time PCR sensitivity and specificity vary based on detection methods as well as laboratory to laboratory practise [118, 123, 124].

## 4. *Helicobacter pylori* Detection Methods 

Various methods have been developed to detect* H. pylori* infection, whereas the gold standard detection remains debatable [125]. In* H. pylori* epidemics study, the sensitivity of tests varies for the direct test (histopathology/IHC or rapid urease test); many noninvasive tests are developed which are called indirect test (serology, UBT, and SAT) to determine infection status [126].

### 4.1. Serological Test

Serological testing using patients' blood and ELISA techniques to detect IgG, IgM, and IgA for* H. pylori* have been developed. Serological testing has uniformly high sensitivity (90 to 100%), variable specificity (76 to 96%), and the accuracy range between 83 and 98%; however, it does not discriminate between current infection or recent exposures [127, 128]. Serological tests require validation at the local level, which is impractical in routine practice. Moreover, serologic findings in both the children and adults are conflicting, and the cut-off is not shown to be accurate in many studies [129, 130]. Serological testing is accurate in low prevalence regions where less than 20% of the population are affected. In those patients where the gastric lining has not changed to the precancerous form of intestinal metaplasia, neither biopsy nor Urea Breath Tests can be used as there are very few bacteria present [131, 132]. Moreover, serial serology from antibody concentrations can be used as follow-up after treatment of* H. pylori* [133].

### 4.2. Urea Breath Test (UBT)

UBT measures C13 carbon dioxide in breath after ingesting C-13-labelled urea [134]. This test is approved by FDA, USA. This can be used for the individuals aged 3 years or older. The cost of UBT is more than serological or stool antigen testing and UBT can be used both as a diagnostic tool and in efficacy of treatments [131, 132].

### 4.3. Stool Antigen Test (SAT)

Antigens released from the wall of the stomach can be detected in SAT through ELISA. Detection of antigen only occurs if* H. pylori* is present and this shows active infection [135]. Similar to the UBT, the SAT can be used both as a diagnostic tool and in efficacy of treatments [125]. This is also an FDA-approved test and SAT is recommended by the ACG and the AGA [136, 137].

## 5. EBV Infection to Gastric Epithelial Cells

Latency and reactivation are the hallmarks of EBV which is a ubiquitous and potentially oncogenic human herpes virus [87]. EBV was discovered in 1964 in patients with Burkett's lymphoma (BL) [138]. Initially, it was assumed that it infects only B-cells; later, it was also found in nasopharyngeal epithelial cells [139], liver cells [136], stomach epithelial cells [91], brain cells [137], and so forth.

### 5.1. Low Tropism of EBV Infection through Oral Route to Mouth Oropharyngeal Epithelial Cells

Due to difficulties in establishing reproducible and robust infection* in vitro*, it is very difficult to simulate a real understanding of EBV pathology [114]. Most of the studies suggested that EBV may be transcytosed via EBV+ IgA complex through the oral epithelium, oropharynx bidirectional, from apical membranes to the basolateral and vice versa [140]. This EBV transmigration potentially contributes to initial EBV penetration into B-cells that starts the systemic infection. EBV secretion may occur into saliva in EBV-infected individuals [114, 141]. However, EBV could not be detected in oropharyngeal in the primary stage of infection in the process of transcytosis [142].

### 5.2. EBV Has High Tropism for B Lymphocytes

EBV interacts with naive or memory B lymphocytes in Waldeyer's ring. Waldeyer's ring is situated in lymphoid tissue and surrounds the oropharynx [143]. EBV have a high affinity for the B-cell and complement receptor type 2 (CR2) or CD21 present on the surface of B-cell facilitate the attachment of EBV envelope glycoprotein gp350/220 to B-cell [144, 145]. Following attachment, internalization of EBV occurs in the cells via the endocytosis. Further, this fusion of EBV envelopes proteins and B-cells triggers interaction of another envelopes glycoproteins, that is, gp42. Gp42 interact with HLA class II which is present on B-cells and make a core fusion complex gh/gL/gp42 and further make internalization process [146, 147].

Fate of EBV-infected B-cells depends on their niche as these cells may initiate proliferation or they can reach the memory compartment. EBV establishes a latency and expresses some limited sets of genes if B-cells reach memory compartment [144, 145]. B-cell infection mostly causes latency (I, II, III) [86, 148]; however, freshly isolated B-cells from an EBV-infected tumor lead to transformation and reactivation* in vitro* [149, 150].* In vivo* study suggested that EBV-infected B-cells cause infectious mononucleosis with an incubation period of 30–50 days [81, 121]. Several studies suggested that it is difficult to determine EBV DNA in epithelial cells during primary infection. It is debatable how epithelial cells spread the virus to precede infection of B lymphocytes. Moreover, later EBV-related severe disease shows virus amplification in epithelial cells before shedding in saliva which at least gives some evidential support as virus shed almost daily in the saliva of carriers has the glycoprotein composition of the virus made in an epithelial cell rather than a B-cells [122, 151].

Infected B-cells reach the circulation, and some B-cells may also go to transformation [152, 153]. Cytotoxic T lymphocytes response occurs for the B-cells and mostly this process is due to latent B-cells infection. However, terminal differentiation of B cell occurs through immune response [154]. Infected memory B-cells may reach the site of immune response and further divide into plasma and memory cells. This process initiates reactivation of EBV into lytic cycle in B-cells and this causes more infection to noninfected B-cells and hence replenishes EBV-infected B-cells latent reservoir. This establishes a cycle of persistence in the life of a healthy carrier [155–158]. It is assumed that lytic release of the virus has a high tropism to epithelial cells than B-cells [159].* In vitro* studies suggested that EBV loaded B-cells or B-cells fragments have a high rate of infectivity to epithelial cells [160, 161].

### 5.3. Lytic Release of EBV from B-Cells Has a High Tropism to Infect Other Epithelial Cells

Terminal differentiation of infected memory cells triggers EBV lytic replication [154]. This can occur in any parts/organs of the host where the infected memory cells travel and EBV spread through cell to cell contact [162, 163]. A study suggested that undifferentiated basal epithelial cells support latent EBV infection, while differentiation of epithelial cells promotes lytic reactivation [157]. A direct coculture experiment of epithelial cells with EBV-producing Akaka cells shows that cell-cell contact is required for the EBV entry to epithelial cells. An increase of infection efficiency was observed up to 1,000 times as compared to the only viral supernatant harvested from EBV-producing cells [158, 160].* In vitro* experiment suggested that complement receptor 2 (CR2) was not behind the epithelium infection for EBV, as CR2 expression was not detected in most of the infected epithelial cells [164]. Hence, it is thought to be triggered by binding of epithelial cells integrins *α*v6 or *α*v8 to viral glycoproteins gH/gL [165]. However, these integrins receptors present on epithelial cells show a week affinity with EBV compared to CR2, and hence a cell to cell contact is necessary for the attachment of the receptors of virus released from B-cells to the epithelial cells [158, 160].

**Figure 2:**
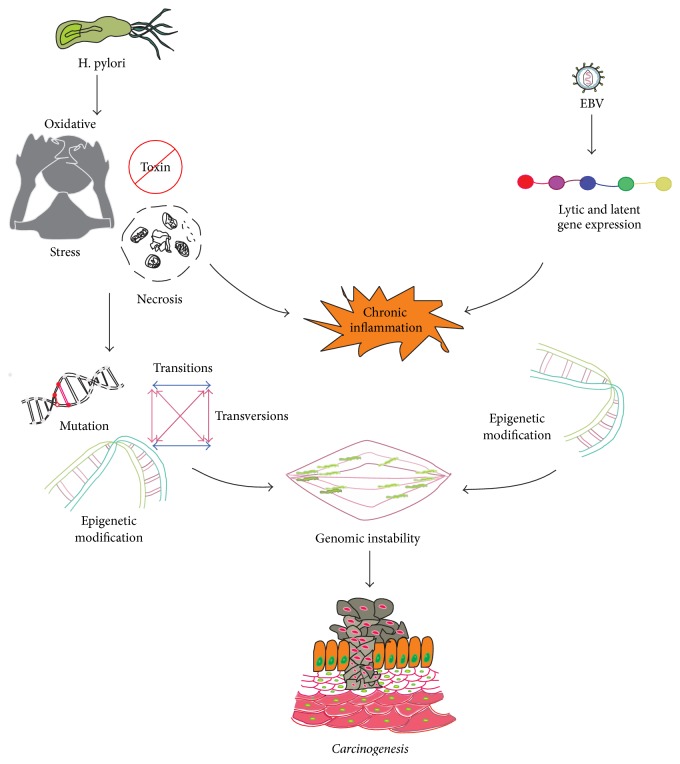
Mechanisms of EBV and* H. pylori* coinfection in gastric epithelial cells. A detailed illustrative mechanism demonstrated in gastric epithelial cells.* H. pylori* infection leads to oxidative stress, toxin, and necrosis in cells. These reactions further lead to chronic inflammation, epigenetic modification, and mutation. All these alterations led to genomic instability. EBV infection leads to the expression of lytic and latent genes of EBV. These viral genes regulated epigenetic modification and chronic inflammation. Further these EBV derived mechanisms lead to genomic instability. Finally, genomic instability is one of the potent sources of carcinogenesis.

Another study of the coinfection suggested that CD21 receptor on epithelial cells plays an important role in infecting epithelial cells from EBV-producing B-cells. EBV induces a strong adhesion between B-cells and epithelial cells through activation of CD21 [166]. In a coculture experiment of EBV infected B-cells prelabelled with mABs to its cell surface and epithelial cells shows interaction of EBV glycoprotein gp350 with the CD21 complex members, CD21, CD81, and CD19, between B-cell and epithelial cell synapse [166]. Members of tetraspanins, CD82 and CD63, members of integrin's family LFA-1, integrin *β*1, CD11b, and integrin *α*v*β*6, and members of Ig superfamily ICAM-1 and CD48 also show interaction of EBV glycoproteins and CD21 [166]. Virus genome integrates into host epithelial cell genome and amplifies with it [162]. EBV establishes a cycle of persistence in a healthy human and starts to be released in saliva or infects more B-cells. Thus, EBV spread throughout the superbasal epithelium and express latent as well as lytic proteins (Figure 2) [163, 167].

## 6. *Helicobacter pylori* Infection in Epithelial Cells

*H. pylori* infection spreads from contaminated food or may also be transferred from faces to the mouth [70]. The bacteria neutralize stomach acids and cause gastric ulcer when they penetrate the gastric mucous lining [168]. Two types of* H. pylori* may be found in the gastric or mucosal lining: coccoid type and helical type [169, 170]. Helical type of* H. pylori* may be transformed into coccoid type. Coccoid type is less vulnerable with low antigenicity and less vulnerable in gastric lining with low production of cytotoxic protein products (CagA), arginase RocF, tumor necrosis factor-*α* (TNF-*α*), and others [171]. This makes* H. pylori* escape from immune response more easily [169, 170, 172, 173]. Nearly, 20% of* H. pylori* in the stomach lining adhere to the epithelial cells surface while the rest are attached through the cell to cell junction. Few numbers of* H. pylori* bacteria are also found in deeper intercellular space [174]. Autotransporter proteins present on* H. pylori* surface, BabA, SabA, AlpA, AlpB, HopZ, OipA, and others, facilitate the adherence to the epithelial surface [174–181]; however, no individual protein is found essential [182, 183]. Additionally, differential expression of these proteins occurs between strains as well as within a single strain. Thus, over time,* H. pylori* acquire a dynamic adaption by alteration in gene expression, inactivation, or recombination (Figure 2) [184, 185].

## 7. **Methylation**

### 7.1. EBV and Methylation

Promoter region hypermethylation in certain genes is frequently seen in EBV-positive GC compared to EBV-negative GC [16, 186]. GC and other most common cancers occur by the genetic and epigenetic changes over an extended period. Methylation is common in cancer and can be divided into two categories, complete genome hypomethylation which causes cancer due to genetic reason [187, 188] and regional hypermethylation that are mostly caused by infection or long-term inflammation [187, 189, 190]. Host cellular machinery plays a more important role and induces aberrant methylation than viral factors [188, 191]. Host cells initiate dense methylation to silence EBV genes but in this process host genes themselves become extensively methylated [192, 193].* H. pylori* are considered as major factors of GC, and aberrant methylation is also the hallmark of* H. pylori*-related GC [194]. Hypermethylation has been linked to* H. pylori*-related gastritis and inflammation [195]. The mechanisms of* H. pylori* induced hypermethylation are unknown and it is also thought that there is possible involvement of ROS/NOS [196]. Though several studies suggest an association of EBV and* H. pylori* coinfection in the occurrence of GC, the mechanism is still unclear [78, 197, 198]. A recently published data in AGS cell line demonstrated that EBV also methylated those host genes which are associated with neutralized CagA toxin of* H. pylori* [199]. Another study suggests that cooperation of EBV gene Zta with* H. pylori* has some positive link to GC [79].

In a study in cancer-related signalling pathways in EBV-associated GC, genes of cell cycle regulation (IGFBP3, CDKN2A, ID2, HSP70, CCND1, and ID4), DNA repair (BRCA1, TFF1), cell adhesion (ICAM1), angiogenesis (HIF1A), and inflammation (COX2) were found deregulated [200]. EBV-specific patterns were observed in CpG island DNA methylation and demethylation for some promoter sequence [201, 202]. The loss of 3 critical tumor suppressor genes, CDH1 (E-cadherin) [198], p73 [203], and CDKN2A (p16) [201], in EBV-associated GC is also seen. EBV-specific CpG island methylation and demethylation were observed by bisulfite DNA sequencing [202]. However, EBV is associated with epigenetic changes of apoptosis (DAPK, BNIP3, FAM3B HRK, IL15RA, MINT31, p16, p73, PTEN, and RASSF1A), cell cycle regulation (APC, p15, p16, p57, and p73), cell proliferation (E-Cadherin, HRASLS, IL15RA, MINT31, NKX3.1, RUNX3, TIMP2, and TIMP3), cell signalling (14-3-3 Sigma, CSPG2, MINT1, MINT2, and PLXND1), cell adhesion (EPHB6, FLNC, FSD, REC8, and CSPG2), migration (EPHB6), interaction (MDGA2, THBS1), DNA repair (HMLH1, MGMT), and many other epigenetic changes (BCL7A, BLU, CHFR, CXXC4, GSTP1, HLTF, HOXA10, IHH, MARK1, MINT25, PAX5-*β*,SCARF2, SSTR1, THBD, and WNT5A) [17, 40, 190, 191, 194, 204–211]. Associations of EBV factors with different host machinery and methylation are listed in Tables 2 and 3.

Aberrant DNA methylations are catalyzed by the enzymes, namely, DNA methyltransferases (DNMTs) [54]. DNMT1, DNMT3A, and DNMT3B are isoforms of DNMTs which maintain the original methylation patterns after replication and target unmethylated CpG islands to initiate methylation [55]. Overexpression of these 3 isoforms was observed in* H. pylori*-related GC [56]. It was reported that CDH1 gene methylation was higher in* H. pylori* associated gastric mucosa than in* H. pylori* negative gastric mucosa [57, 212]. CDH1 is a cell-cell adhesion glycoprotein, which is frequently inactivated in GC. In* H. pylori* induced gastritis, COX2 [213], IL1-*β* [214], IFN-*γ* [215], TNF-*α* [216], NOS 2 [217], and genes associated with the inflammation were found to be highly upregulated [218]. A study suggested upregulation of SMARCD1 protein through miR-490-3p in* H. pylori* associated GC. Further, overexpression of this protein causes oncogenic phenotype expression in* in vitro* and* in vivo* studies [219]. In another study, downregulation of Gastrokine (GKN1) was observed in* H. pylori* associated GC. GKN1 facilitates the restoration and proliferation after gastric epithelial injury and suppresses GC. This study also revealed that GKN1 inversely correlated the expression of DNMT1 and EZH2 (enhancer of zeste homologue 2) [220]. EZH2 is a potential target of many types of cancers [221]. Another study suggested that deregulation of Forkhead box protein (FOX) and methylation was observed in* H. pylori* associated GC. Also, dysregulation of FOXD3 promotes gastric carcinogenesis [222]. Many other genes found upregulated by* H. pylori* are associated with cell cycle progression and proliferation (p14, p16, p21, p27, RAB40C, COX 2, FOS, ERBB2, FGFR2, ABL1, ECOP, JAK2, MYC, MET, SIRT1, PDCD4, TRAF6, GMNN, and CCNE2) [213, 223–242], apoptosis (RECK, SMAD4, TRAIL, MCL1, BIM, XIAP, and PDK1) [243–250], and invasion and metastasis (PTEN, WNT 5a, EDNRA, ROR2, EPB41L3, MMP1, MMP10, HMGA2, ROBO1, TGF-*β*, EZH2, casein kinase 2, and ZEB) [251–262]. Several studies showed that the upregulation of inflammatory cytokines IL1-*β*, NOS 2, and TNF-*α* induced methylation [46–49, 51, 52, 263].* H. pylori* induces oxidative stress, ROS, and RNS which can cause p53 point mutations [264–267]. Nitric oxide (NO) can cause G:T mismatch during DNA synthesis and eventually results in G:C to A:T base transversion and epigenetic modification of tumorigenic genes (Figure 2) [268, 269].

## 8. EBV and* H. pylori* Factors Contributing to the Development of GC

Interaction between EBV and* H. pylori* in host stomach lining may have some synergistic effects in the development of GC. Many genes were found methylated in EBV and* H. pylori* coinfected gastric adenocarcinomas. Most frequently hypermethylated genes include COX 2, DAPK, CDH1, CDKN2A, and hMLH1. These genes are commonly found altered in various cancer types including GC [270]. Further,* H. pylori* positive individuals show a significantly higher EBV DNA load which suggests* H. pylori* role in lytic phase conversion of EBV [271]. Also, EBV DNA load was more in* H. pylori* positive patients than those uninfected with GC [272]. Another study on coinfection suggests that EBV with* H. pylori* induces severe inflammatory responses in the individual and, hence, increases the risk of developing the intestinal type GC [78]. It is thought that there are two possible mechanisms, first an additional inflammatory response in coinfection and increased tissue damaging by both* H. pylori* and EBV [79, 215]. In this scenario, significant elevation was observed in IL-1*β* [273], TNF-*α* [274], and IL-8 [275]. A study in pediatrics patients demonstrated that* H. pylori* infection was not but the presence of EBV, an essential factor for severe inflammation [79]. The second mechanism is based on gene products interaction which is more significant between EBV and* H. pylori*.* In vitro* study found that EBV reactivation occurs by the PLC*γ* signalling pathway and* H. pylori* toxin CagA strongly activates PLC*γ* [237] and also activates several kinases [276]. An ectopic expression on transgenic mice supports the oncoprotein nature of CagA [277–280]. CagA of* H. pylori* and LMP1 and LMP2 of EBV activate NF-*κ*B and MAP kinases, which are well-known pathways of cell survival and proliferation during carcinogenesis [211, 281].* H. pylori* associated oncoprotein CagA triggers an aberrant activation of WNT signalling pathway [282]. WNT signalling pathway activation leads to the activation of CDX1, a downstream gene [283], which reprograms epithelial cells in mucosal lining to acquire stemness properties by inducing SALL4 and KLF5 factors [284]. Another study also suggests that EBV and* H. pylori* transform the stomach epithelium cells and play roles in carcinogenesis [78]. Both pathogens induce common pathways which leads to the activation of transforming factors in stomach epithelial cells by *β*-catenin/TCF-4 signalling pathway [79, 285]. In another study, an association between EBV and* H. pylori* copositivity was shown and significant infiltration in premalignant lesions in GC was observed [78]. A study by Szkaradkiewicz et al. suggested that BcL2 expression was higher in EBV and* H. pylori* associated GC; thus, excessive overexpression may be the result of coinfection [286]. Several studies also revealed that PCDH10 (protocadherin 10) is calcium dependent cell adhesion molecule which suppresses tumor in gastric epithelial hypermethylated in* H. pylori* associated GC and EBV-infected individual [287–289]. SWI/SNF remodelling complex which is commonly observed in GC is found associated with both pathogens, EBV and* H. pylori* [290]. A recent study suggested that host protein SHP 1 interacts with* H. pylori* CagA protein and dephosphorylates CagA, thus preventing oncogenic activity of CagA. However, EBV coinfection causes methylation of host SHP 1 and prevents its dephosphorylation activity of CagA and thus may increase oncogenic potential of CagA [199]. Further, a study suggested that both EBV and* H. pylori* coinfection were ominously more dominant in intestinal ulcer patients compared to GERD and dyspepsia patients [291].* H. pylori* positive patients show increased anti-EBV IgG titre which suggests* H. pylori* role in augmenting EBV DNA load and higher immune responses [291]. However, some study is also available which suggested that* H. pylori* attenuated TGF-*β* expression which reactivates EBV lytic phase and might play a role in preventing EBV lytic reactivation and preventing GC [292]. Therefore, the mechanism of coexistence for* H. pylori* and EBV must be studied to find the probable and potential pathogenic roles for both pathogens.

## 9. Future Direction

To date, mostly clinical findings explicitly described the EBV and* H. pylori* coinfection in GC. Moreover, how these pathogens target host factors and downstream pathways is still unexplored. Therefore, a detailed study which could potentially uncover the mechanism of EBV and* H. pylori* in the progression of GC could be interesting to peruse. How* H. pylori* antigens interacted with EBV antigens could be interesting to explore and helps in the understanding of progression of aggressive GC. Why only few cells from host are targeted by* H. pylori* and EBV is also critical to understand.

## Figures and Tables

**Table 1:** 

Anti-VCA IgM	Anti-VCA IgG	Anti-EBNA 1 IgG	Anti-EA (D) IgG	Interpretation
−	−	−	−	No infection
+	+	−	−	An early and primary infection
− or +	+	−	+	An active infection
−	+	+	−	A past infection
−	+	+	+	May indicate reactivation of virus, lytic

Serological results and most likely interpretation: VCA: Viral Capsid Antigen, IgM: immunoglobulin type M, IgG: Immunoglobulin type G, EBNA 1: Epstein–Barr nuclear antigens 1, EA (D): Early Antigen D.

**Table 2:** 

EBV gene	Cellular response	Reference
EBNAs, BALF1, EBERs, BARTs	Tumor growth and metastasis	[36–38]
LMP 1, EBNAs	Angiogenesis	[38, 39]
LMP1, BARTs	Invasion, metastasis	[36, 40]
BARTs, EBNAs, LMPs	ECM remodelling	[39, 41, 42]
EBNAs, LMPs, EBERs, BARTs	Cell migration	[43–45]
EBNAs, LMPs, Zta, BARTs	Stemness	[41, 42]

EBNAs: Epstein-Barr nuclear antigens, BALF1: LMP1: latent membrane protein 1, EBERs: Epstein-Barr virus-encoded small RNAs, BARTs: Bam HI A rightward transcripts, LMPs: latent membrane proteins, and Zeta: protein encoded by BZLF1.

**Table 3:** 

EBV gene	Host gene interaction	Reference
LMP 1	CDH1	[42, 43]
LMP2A	PTEN, STAT3	[46–48]
EBERs	IGF-I	[49, 50]
LMP1, LMP2A	DNMT1, DNMT3b	[51, 52]
BARF1	Cyclin D, NFkB	[49, 53]
Zta	Acetyl-transferase protein CBP, EGR1	[54–57]

LMP: latent membrane protein, EBERs: Epstein-Barr virus-encoded small RNAs, BARF1: Bam HI-A rightward frame 1, CDH1: Cadherin 1, PTEN: phosphatase and tensin homolog, IGF1: insulin-like growth factor 1, DNMT1: DNA methyltransferase 1, DNMT3b: DNA methyltransferase 3b, and EGR 1: early growth response gene 1.
